# Assessing the relationship between burnout syndrome and irritable bowel syndrome among medical health providers and medical students in Saudi Arabia

**DOI:** 10.25122/jml-2022-0242

**Published:** 2023-02

**Authors:** Mohammed Attieh Alzahrani, Hassan Ali Alamri, Mohammed Aedh Alshehri, Msawed Muhammed Ayyashi, Saeed Ali Alqarni, Salem Hassan Alshehri, Mohammed Salem Alshehri, Majed Musfer Alqahtani, Nader Hasan Alasmari, Abdulmalik Mohammed Alsabban, Abdulaziz Saad Alshahrani

**Affiliations:** 1 Internal Medicine, College of Medicine, King Khalid University, Abha, Saudi Arabia; 2 Gastroenterology Department, Assir Central Hospital, Abha, Saudi Arabia; 3 Internal Medicine, King Abdullah Hospital, Bisha, Saudi Arabia; 4 Department of Medicine, College of Medicine, King Khalid University, Abha, Saudi, Arabia; 5 King Abdulaziz Medical City, Jeddah, Saudi Arabia; 6 Internal Medicine, College of Medicine, Najran University, Saudi Arabia; 7 Department of Medicine, King Khalid Hospital, Najran, Saudi Arabia

**Keywords:** burnout, irritable bowel syndrome, prevalence, severity, healthcare staff

## Abstract

Burnout syndrome, characterized by chronic unmanageable workplace stress, has been linked to lower gastrointestinal disorders, including irritable bowel syndrome. However, the relationship between burnout syndrome and irritable bowel syndrome among medical health providers and medical students in Saudi Arabia has not been fully explored. This cross-sectional correlational study was conducted in Southern Saudi Arabia from 2021 to 2022 and involved 931 medical health providers and medical students who completed an electronic questionnaire. The study assessed the presence and severity of burnout and irritable bowel syndrome and examined their relationship. Burnout syndrome was evaluated using the Maslach Burnout Inventory-Student Survey (MBI-SS), while irritable bowel syndrome criteria and severity were assessed using validated tools. The study found that 85% of medical health providers and medical students experienced high levels of burnout and irritable bowel syndrome severity, with physicians and nurses mainly affected. Occupational exhaustion was high in 44.4% of participants, while depersonalization was high in 53% of participants. Personal accomplishment was low in 73.5% of participants. Mild, moderate, and severe irritable bowel syndrome was reported in 25.6%, 23.8%, and 12% of participants, respectively. The study highlights a significant association between burnout syndrome and irritable bowel syndrome severity among medical health providers and medical students in Saudi Arabia. These findings underscore the importance of developing effective interventions to prevent and manage burnout syndrome and related health issues among healthcare professionals and medical students in the region.

## INTRODUCTION

The physical and mental health of healthcare providers is crucial to ensuring the safety and quality of patient care. However, burnout among healthcare professionals has been a persistent issue for many years [1,2]. Burnout syndrome is a condition that develops as a result of prolonged and unmanageable workplace stress, and it has been prevalent throughout the careers of healthcare providers [3-7]. Furthermore, it is a condition typically characterized by three main dimensions: emotional exhaustion, depersonalization or feelings of mental distance from one's job, and reduced personal accomplishment or a sense of reduced professional efficacy [8].

Irritable bowel syndrome (IBS) is a well-defined disorder characterized by abdominal discomfort or pain associated with changed bowel habits for at least three days per month in the previous three months [9]. Common symptoms include abdominal pain in the form of cramping sensations, diarrhea, constipation, or alternating diarrhea and constipation [10]. Treatment goals for IBS typically focus on symptom relief and improving patients’ quality of life [11,12].

**Figure 1:**
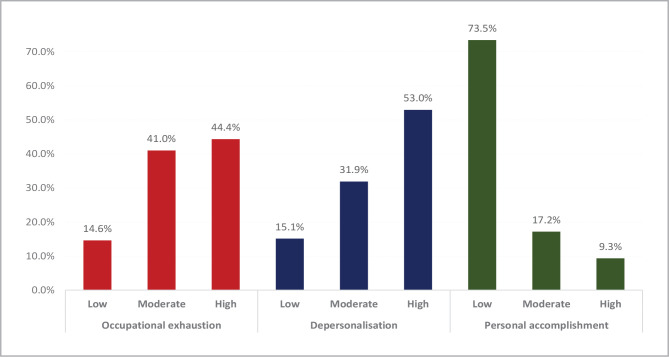
Burnout level among medical health providers and medical students in Saudi Arabia.

Healthcare providers face different stressors in their work environments as they carry out their responsibilities to provide health and medical care to patients [13]. Mental health problems are particularly prevalent among healthcare providers, and nursing is ranked 27th out of 130 high-stress occupations in terms of the prevalence of health disorders, according to the National Institutes of Health [14]. The demands and pressures of providing medical care can result in high-stress levels for healthcare providers. This stress can have detrimental effects on the quality of care they provide, as well as their attitudes and behaviors toward their patients (Figure 1) [15, 16]. All these factors put healthcare providers at higher risk of burnout and other medical conditions, including gastrointestinal disorders, mainly bowel dysfunction. The current study aimed to assess the severity of IBS and burnout syndrome among medical health providers and medical students in Saudi Arabia. We also investigated the relationship between burnout and irritable bowel syndrome among these groups.

## MATERIAL AND METHODS

A correlational cross-sectional study was conducted in Abha, Southern Saudi Arabia, between 2021-2022, targeting healthcare providers and medical students. Data were collected through an electronic questionnaire developed by the researchers after conducting a literature review of relevant articles and consulting experts at King Khalid University. The questionnaire covered participants’ age, job titles, and work settings.

Maslach Burnout Inventory-Student Survey (MBI-SS) was used to assess burnout among study participants. MBI -SS items are scored using 7-level frequency ratings from "never" to "daily." The MBI-SS is composed of three scales: emotional exhaustion (9 items), depersonalization (5 items), and personal achievement (8 items). Each scale measures its unique dimension of burnout [17, 18].

IBS criteria and severity assessment, as well as gastroesophageal reflux disease (GERD) symptoms, were used to assess irritable bowel syndrome with a total score of 500 for the main 5 sentences as a score between less than 75 indicates mild IBS; 175 ≤ 300 indicates moderate IBS; > 300 indicates severe disease [9, 19]. To ensure the validity and clarity of the study questionnaire, a panel of three experts at King Khalid University reviewed it and made necessary modifications. The approved questionnaire was uploaded online using social media platforms by the researchers and their contacts.

### Data analysis

The collected data was reviewed, coded, and entered into IBM SPSS version 22 (SPSS, Inc. Chicago, IL) for analysis. All statistical analyses were performed using two-tailed tests, with a P-value less than 0.05 considered statistically significant. To assess burnout, Maslach Burnout Inventory-Student Survey (MBI-SS) scores were calculated for different domains and categorized into low, moderate, and high levels according to documented scale cut-off points [1, 2]. IBS criteria and severity were assessed using validated tools, and the severity score was categorized as mild, moderate, or severe IBS [3,4]. Descriptive analyses were conducted using frequency distribution with percent to assess participants' data, burnout levels, and severity of IBS. Cross-tabulation was performed to assess the distribution of participants' burnout levels and IBS severity scores by job title. Additionally, the relationship between burnout levels and IBS severity among healthcare professionals and students was examined. The significance of these relationships was assessed using the Pearson chi-square test and the exact probability test for small frequency distributions.

## RESULTS

931 out of 1031 medical health providers and medical students fulfilling the inclusion criteria completed the study questionnaire. Participants' ages ranged from 18 to 59 years old, with a mean age of 25.2 ±11.9 years old. 402 participants (43.2%) were physicians, including doctors, dentists, and pharmacists, while 265 (28.5%) were nurses. Medical students accounted for 197 (21.2%) participants, and 67 (7.2%) were paramedical professionals. In terms of work setting, 443 (47.6%) participants worked at hospitals, 364 (39.1%) at universities, and 124 (13.3%) worked at primary healthcare centers (Table 1).

**Table 1:** Personal characteristics of medical health providers and medical students.

Characteristics	No	%
**Age in years**		
<20	10	1.1%
20–29	588	63.2%
30–39	224	24.1%
40–49	93	10.0%
50+	16	1.7%
Mean age: 25.2±11.9 years old		
**Job title**		
Physician	402	43.2%
Nurse	265	28.5%
Paramedical personnel	67	7.2%
Student	197	21.2%
**Setting**		
Hospital	443	47.6%
Primary health care	124	13.3%
University	364	39.1%

We found that a significant proportion of participants reported high levels of occupational exhaustion (44.4%) and depersonalization (53%). Conversely, 136 (14.6%) participants reported low levels of occupational exhaustion, and 141 (15.1%) participants reported low levels of depersonalization. Furthermore, most participants reported low levels of personal accomplishment (73.5%), with only a small proportion reporting high levels (9.3%).

Among physicians, 48% reported significantly higher occupational exhaustion compared to 44.9% of nurses, 44.8% of paramedical, and 36% of students (P=.048). Depersonalization was higher among nurses (59.6%) than physicians (51%), paramedical (49.3%), and students (49.2%). However, this difference was not statistically significant (P=.143). In terms of personal accomplishment, 77% of the nurses reported low levels in comparison to 75.6% of students, 71.4% of physicians, and 65.7% of paramedical with no statistical significance (P=.376) (Table 2).

**Table 2:** Comparing burnout rates among participants by job title.

Burnout	Job title								P-value
	Physician		Nurse		Paramedical		Student		
	No	%	No	%	No	%	No	%	
**Occupational exhaustion**									
Low	49	12.2%	45	17.0%	13	19.4%	29	14.7%	.048*
Moderate	160	39.8%	101	38.1%	24	35.8%	97	49.2%	
High	193	48.0%	119	44.9%	30	44.8%	71	36.0%	
**Depersonalization**									
Low	60	14.9%	33	12.5%	15	22.4%	33	16.8%	.143
Moderate	137	34.1%	74	27.9%	19	28.4%	67	34.0%	
High	205	51.0%	158	59.6%	33	49.3%	97	49.2%	
**Personal accomplishment**									
Low	287	71.4%	204	77.0%	44	65.7%	149	75.6%	.376
Moderate	75	18.7%	37	14.0%	14	20.9%	34	17.3%	
High	40	10.0%	24	9.1%	9	13.4%	14	7.1%	

P – Pearson X^2^ test; * – P<0.05 (significant).

**Figure 2:**
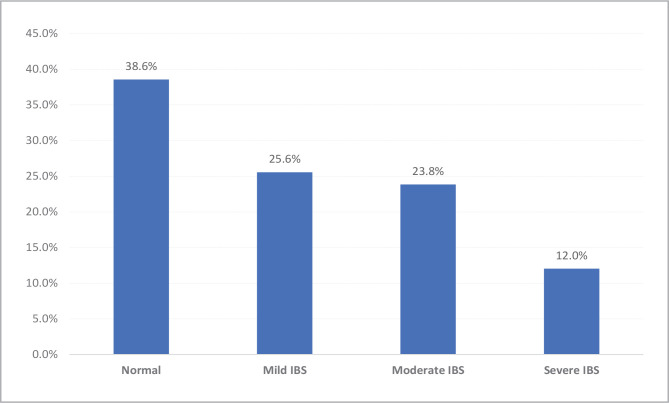
Irritable bowel syndrome severity among medical health providers and medical students in Saudi Arabia.

359 (38.6%) participants did not meet the clinical criteria for IBS. Mild IBS was present in 238 (25.6%) participants, while 222 (23.8%) had moderate IBS, and 112 (12%) had severe IBS. Mild to severe IBS was found in 61.9% of physicians, compared to 63% of nurses, 62.7% of paramedical, and 57.9% of students, with no significant statistical difference (P=.340) (Figure 2, Table 3).

**Table 3:** Distribution of IBS severity by participants’ job title.

IBS severity	Job title								P-value
	Physician		Nurse		Paramedical		Student		
	No	%	No	%	No	%	No	%	
**No IBS**	153	38.1%	98	37.0%	25	37.3%	83	42.1%	.340
**Mild**	92	22.9%	70	26.4%	17	25.4%	59	29.9%	
**Moderate**	100	24.9%	64	24.2%	17	25.4%	41	20.8%	
**Severe**	57	14.2%	33	12.5%	8	11.9%	14	7.1%	

P – Pearson X^2^ test.

A high proportion of participants (89.5%) had abnormal bowel movements, while 72.4% had hard stools. The consistency of stools was described as very thin, "like string" among 62.1% of participants, in small pieces among 63.9%, mushy "like porridge" among 59.2%, and watery among 58.4%. Furthermore, nearly half (49.3%) of the participants reported the feeling of incomplete bowel evacuation after defecation. Additionally, 40.7% reported the need to hurry/rush to the toilet for bowel movements, and 37.9% experienced straining during bowel movements. Nearly one-fifth (19.2%) of the participants reported passing bloody stools (Table 4).

**Table 4:** Bowel habits among medical health providers and medical students of Saudi Arabia.

Bowel habits	No	%
**Do you typically have bowel movements that you consider to be normal, without any unusual or concerning features?**		
Often	495	53.2%
Occasionally	338	36.3%
Never	98	10.5%
**Do you ever experience difficulty passing stool, such as having to strain or feeling like the stool is hard?**		
Often	191	20.5%
Occasionally	483	51.9%
Never	257	27.6%
**Do you ever have bowel movements that are very thin, similar to a string?**		
Often	174	18.7%
Occasionally	404	43.4%
Never	353	37.9%
**Do you ever pass bowel movements that are small in size and broken into pieces?**		
Often	199	21.4%
Occasionally	396	42.5%
Never	336	36.1%
**Do you ever have bowel movements that are mushy in texture, similar to porridge?**		
Often	160	17.2%
Occasionally	391	42.0%
Never	380	40.8%
**Do you ever have bowel movements that are loose or watery in consistency?**		
Often	155	16.6%
Occasionally	389	41.8%
Never	387	41.6%
**Do you ever pass mucus (or slime or jelly) along with your bowel movements?**	352	37.8%
**Do you ever pass blood along with your bowel movements?**	179	19.2%
**Do you ever feel the need to hurry or rush to use the toilet for a bowel movement?**	379	40.7%
**Do you ever experience the need to strain or push to have a bowel movement?**	353	37.9%
**Do you ever feel like you haven't completely emptied your bowel after having a bowel movement?**	459	49.3%

There was a significant relationship between high levels of occupational exhaustion and the severity of IBS, with 81.4% of participants with high occupational exhaustion experiencing mild to severe IBS, compared to 57.9% of those with a moderate degree and 41.2% of those with a mild degree of occupational exhaustion (P=.001). Participants with high levels of depersonalization also showed a higher prevalence of IBS, with 66.9% of participants experiencing IBS of varying severity, compared to 57.2% of those with moderate depersonalization and 51.1% of those with a mild degree of depersonalization (P=.001). However, there was no significant relationship between personal accomplishment and IBS severity among study participants (P=.460) (Table 5).

**Table 5:** The relationship between burnout and irritable bowel syndrome among medical health providers and medical students in Saudi Arabia.

Burnout	IBS severity								P-value
	No IBS		Mild		Moderate		Severe		
	No IBS	%	No	%	No	%	No	%	
**Occupational exhaustion**									
Low	80	58.8%	30	22.1%	22	16.2%	4	2.9%	.001*
Moderate	161	42.1%	117	30.6%	81	21.2%	23	6.0%	
High	118	28.6%	91	22.0%	119	28.8%	85	20.6%	
**Depersonalization**									
Low	69	48.9%	32	22.7%	28	19.9%	12	8.5%	.001*
Moderate	127	42.8%	89	30.0%	56	18.9%	25	8.4%	
High	163	33.1%	117	23.7%	138	28.0%	75	15.2%	
**Personal accomplishment**									
Low	252	36.8%	176	25.7%	175	25.6%	81	11.8%	.460
Moderate	68	42.5%	42	26.3%	30	18.8%	20	12.5%	
High	39	44.8%	20	23.0%	17	19.5%	11	12.6%	

P – Pearson X^2^ test; * – P<0.05 (significant).

## DISCUSSION

Irritable bowel syndrome (IBS) is a common functional gastrointestinal disorder affecting the general population that can lead to reduced work productivity, impaired quality of life, and a high burden on those affected [20]. Despite the considerable body of literature assessing the prevalence and impact of burnout and IBS among healthcare workers, we are not aware of any prior research conducted in Saudi Arabia that has specifically examined the potential relationship between the degree of burnout and IBS severity among healthcare providers.

The study showed that the vast majority of healthcare providers had a moderate to a high degree across all dimensions of burnout, which exceeded three-quarters for all dimensions. More than half of the healthcare providers reported high levels of depersonalization (53%), and less than half (44.4%) had a high degree of occupational exhaustion. Also, low personal accomplishment was detected among three-quarters of the study group (73.5%), while only 9% had a high level of personal accomplishment. High levels of occupational exhaustion and depersonalization were significantly more frequent among physicians and nurses than paramedical staff and students. This finding may be explained by the higher workload and increased patient interaction experienced by physicians and nurses compared to technicians and other staff members who primarily perform logistic functions. The high levels of burnout observed among healthcare staff in the current study are consistent with previous research findings. A report by the Agency for Healthcare Research and Quality revealed that burnout affects a significant proportion of healthcare staff, ranging from 10-70% among nurses and 30-50% among physicians, nurse practitioners, and physician assistants [21]. Similarly, a study by Wang J *et al*. found that 65.6% of healthcare professionals caring for patients with prolonged disorders showed burnout (55.2% physicians and 82.9% nurses) [22]. The Maslach Burnout Inventory scores showed that emotional exhaustion and depersonalization were correlated with age, gender, occupation, marital status, years of practice, and education. Furthermore, Glossaries *et al*. and Leonardi *et al*. estimated that many healthcare providers suffer from moderate to low levels of burnout [23,24].

Regarding IBS, the current study showed that about two-thirds (61.4%) of the healthcare providers complained of IBS, which was mild among 1 out of each 4 participants (25.6%) and severe among 12%. Most (89.5%) participants reported abnormal bowel movements, and three-quarters (72.4%) experienced hard stools. Participants reported a variety of abnormal bowel movements, including thin or string-like motions in 62.1%, small pieces in 63.9%, mushy or porridge-like in 59.2%, and watery in 58.4%. Additionally, nearly half (49.3%) reported a sensation of incomplete bowel evacuation after defecation, 40.7% reported the need to rush to the toilet for bowel movements, and 37.9% experienced difficulty and straining during defecation. Approximately one-fifth (19.2%) of participants reported passing bloody stools, and nearly one-third (33%) passed mucus with their motions. These findings were consistent with Jafri W *et al*., who reported that the predominant symptom of IBS was abdominal pain (87.8%) which increased post-prandially [25]. Tosun O. *et al*. studied 44 healthcare professionals with IBS and found that 13.5% had alternate bowel habits, 29.5% had constipated-dominant IBS, and 57% had diarrhea-dominant IBS [20].

Our findings showed a significant relationship between higher levels of occupational exhaustion and depersonalization with more severe degrees of IBS among healthcare providers. Conversely, personal accomplishment showed no significant relation with IBS severity. This relationship is supported by Hod K *et al*., who found that burnout, but not job strain, was associated with the prevalence of IBS in working adults, consistent with other study findings conducted by Cholongitas E *et al*. [26,27].

## CONCLUSION

The results of this study suggest that healthcare providers, particularly physicians, and nurses, have high levels of burnout and IBS severity. Furthermore, the study found burnout syndrome is associated with GI symptoms and IBS severity. These findings have important implications, as reducing stress through training healthcare providers in coping strategies may alleviate GI symptoms, including IBS, and improve overall well-being. It is important to note that the relationship between burnout and IBS is bidirectional, and addressing one may positively affect the other. These findings underscore the importance of addressing burnout among healthcare providers to improve both their mental and physical health, as well as the quality of care provided to patients. Further research is needed to identify effective interventions for addressing burnout and its associated physical symptoms in healthcare providers.
